# Simulating lightning NO production in CMAQv5.2: performance evaluations

**DOI:** 10.5194/gmd-12-4409-2019

**Published:** 2019

**Authors:** Daiwen Kang, Kristen M. Foley, Rohit Mathur, Shawn J. Roselle, Kenneth E. Pickering, Dale J. Allen

**Affiliations:** 1 Center for Environmental Measurement and Modeling, U.S. Environmental Protection Agency, Research Triangle Park, NC 27711, USA; 2 Department of Atmospheric and Oceanic Science, University of Maryland, College Park, MD, USA

## Abstract

This study assesses the impact of the lightning nitric oxide (LNO) production schemes in the Community Multiscale Air Quality (CMAQ) model on ground-level air quality as well as aloft atmospheric chemistry through detailed evaluation of model predictions of nitrogen oxides (NO_*x*_) and ozone (O_3_) with corresponding observations for the US. For ground-level evaluations, hourly O_3_ and NO_*x*_ values from the U.S. EPA Air Quality System (AQS) monitoring network are used to assess the impact of different LNO schemes on model prediction of these species in time and space. Vertical evaluations are performed using ozonesonde and P-3B aircraft measurements during the Deriving Information on Surface Conditions from Column and Vertically Resolved Observations Relevant to Air Quality (DISCOVER-AQ) campaign conducted in the Baltimore– Washington region during July 2011. The impact on wet deposition of nitrate is assessed using measurements from the National Atmospheric Deposition Program’s National Trends Network (NADP NTN). Compared with the Base model (without LNO), the impact of LNO on surface O_3_ varies from region to region depending on the Base model conditions. Overall statistics suggest that for regions where surface O_3_ mixing ratios are already overestimated, the incorporation of additional NO from lightning generally increased model overestimation of mean daily maximum 8 h (DM8HR) O_3_ by 1–2 ppb. In regions where surface O_3_ is underestimated by the Base model, LNO can significantly reduce the underestimation and bring model predictions close to observations. Analysis of vertical profiles reveals that LNO can significantly improve the vertical structure of modeled O_3_ distributions by reducing underestimation aloft and to a lesser degree decreasing overestimation near the surface. Since the Base model underestimates the wet deposition of nitrate in most regions across the modeling domain with the exception of the Pacific Coast, the inclusion of LNO leads to reduction in biases and errors and an increase in correlation coefficients at almost all the NADP NTN sites. Among the three LNO schemes described in Kang et al. (2019), the hNLDN scheme, which is implemented using hourly observed lightning flash data from National Lightning Detection Network (NLDN), performs best for comparisons with ground-level values, vertical profiles, and wet deposition of nitrate; the mNLDN scheme (the monthly NLDN-based scheme) performed slightly better. However, when observed lightning flash data are not available, the linear regression-based parameterization scheme, pNLDN, provides an improved estimate for nitrate wet deposition compared to the base simulation that does not include LNO.

## 1 Introduction

The potential importance of nitrogen oxides (NO_*x*_;NO_*x*_ = NO + NO_2_) produced by lightning (LNO_*x*_) to regional air quality was recognized more than 2 decades ago (e.g., Novak and Pierce, 1993), but, in part due to the limited understanding of this NO_*x*_ source (Schumann and Huntrieser, 2007; Murray, 2016; Pickering et al., 2016), LNO_*x*_ emissions have only been added to regional chemistry and transport models during the last decade (e.g., Allen et al., 2012; Kaynak et al., 2008; Koshak et al., 2014; Smith and Mueller, 2010; Koo et al., 2010). Since NO and NO_2_ coexist in the atmosphere, it is often collectively referred to as LNO_*x*_; however, the immediate release of lightning flashes is just NO, and the schemes in Kang et al. (2019) also generate NO emissions only, so in this paper it is primarily referred to as LNO. As a result of efforts to reduce anthropogenic NO_*x*_ emissions in recent decades (Simon et al., 2015; https://gispub.epa.gov/air/trendsreport/2018, last access: 2 October 2019), it is expected that the relative contribution of LNO to the tropospheric NO_*x*_ burden and its subsequent impacts on atmospheric chemistry as one of the key precursors for ozone (O_3_), hydroxyl radical (OH), nitrate ${\text{NO}}_{3}^{-}$, and other species will increase in the United States and other developed countries (Kang and Pickering, 2018). The significant impact of LNO on process-based understanding of surface air quality was earlier reported by Napelenok et al. (2008), who found low biases in upper tropospheric NO_*x*_ in the Community Multiscale Air Quality Model (CMAQ) (Byun and Schere, 2006) simulations without LNO emissions made it difficult to constrain ground-level NO_*x*_ emissions using inverse methods and Scanning Imaging Absorption Spectrometer for Atmospheric Cartography (SCIAMACHY) NO_2_ retrievals (Bovensmann et al., 1999; Sioris et al., 2004; Richter et al., 2005). Appel et al. (2011) and Allen et al. (2012) reported that ${\text{NO}}_{3}^{-}$ wet deposition at National Atmospheric Deposition Program (NADP) sites was underestimated by a factor of 2 when LNO was not included.

LNO production and distribution were parameterized initially in global models (e.g., Stockwell et al., 1999; Labrador et al., 2005), relying on the work of Price and Rind (1992) and Price et al. (1997), so that lightning flash frequency was parameterized as a function of the maximum cloud-top height. Other approaches for LNO parameterization include a combination of latent heat release and cloud-top height (Flatoy and Hov, 1997), convective precipitation rate (e.g., Allen and Pickering, 2002), convective available potential energy (Choi et al., 2005), or convectively induced updraft velocity (Allen et al., 2000; Allen and Pickering, 2002). More recently, Finney et al. (2014, 2016) adopted a lightning parameterization using upward cloud ice flux at 440 hPa (based upon definitions of deep convective clouds in the International Satellite Cloud Climatology Project (Rossow et al., 1996)) and implemented it in the United Kingdom Chemistry and Aerosol model (UKCA). With the availability of lightning flash data from the National Lightning Detection Network (NLDN) (Orville et al., 2002), recent LNO parameterization schemes have started to include the observed lightning flash information to constrain LNO in regional chemical transport models (CTMs) (Allen et al., 2012). In Kang et al. (2019), we described the existing LNO parameterization scheme that is based on the monthly NLDN (mNLDN) lightning flash data and an updated scheme using hourly NLDN (hNLDN) lightning flash data in the CMAQ lightning module. In addition, we also developed a scheme based on linear and log-linear regression parameters using multiyear NLDN-observed lightning flashes and model predicted convective precipitation rate (pNLDN). The preliminary assessment of these schemes based on total column LNO suggests that all the schemes provide reasonable LNO estimates in time and space, but during summer months the mNLDN scheme tends to produce the most LNO and the pNLDN scheme the least LNO.

The first study on the impact of LNO on surface air quality using CMAQ was conducted by Allen et al. (2012) and was followed by Wang et al. (2013) with different ways for parameterizing LNO production and different model configurations. In this study, we present performance evaluations using each of the LNO production schemes (mNLDN, hNLDN, and pNLDN) described by Kang et al. (2019) to provide estimates of LNO in CMAQ. In addition to the examination of differences in air quality estimates between these schemes, we compare the model predictions to Base model estimates without LNO and evaluate the estimates from all of the simulations against surface and airborne observations.

Section 2 describes the model configuration, simulation scenarios, analysis methodology, and observational data. Section 3 presents the analysis results, and Sect. 4 presents the conclusions.

## 2 Methodology

### 2.1 The LNO schemes

In air quality models, three steps are involved in generating LNO emissions: (1) the identification of lightning flashes, (2) the production of the total column NO at model grid cells, and (3) the distribution of the column NO into model layers vertically. Three schemes to produce total column LNO emissions are examined in this study: mNLDN – based on monthly mean NLDN lightning flashes and convective precipitation predicted by the upstream meteorological model; hNLDN – directly uses the observed NLDN lightning flashes that are aggregated into hourly values and gridded onto model grid cells; and pNLDN – a linear and log-linear regression parameterization scheme derived using multiyear observed lightning flash rate and model predicted convective precipitation. After total column LNO is produced at model grid cells, it is distributed onto vertical model layers using the double-peak vertical distribution algorithm described in Kang et al. (2019), which also provides detailed description and formulation of all the LNO schemes.

### 2.2 The CMAQ model and simulation configurations

The CMAQ model (Appel et al., 2017) version 5.2 was configured with the Carbon Bond 6 (CB6) chemical mechanism (Yarwood et al., 2010) and the AERO6 aerosol module (Nolte et al., 2015). The meteorological inputs were provided by the Weather Research and Forecasting (WRF) model version 3.8, and the model-ready meteorological input files were created using version 4.2 of the meteorology–chemistry interface processor (MCIP; Otte and Pleim, 2010).

The modeling domain covers the entire contiguous United States (CONUS) and surrounding portions of northern Mexico and southern Canada, as well as the eastern Pacific and western Atlantic oceans. The model domain consists of 299 north–south grid cells by 459 east–west grid cells utilizing 12 km × 12 km horizontal grid spacing, 35 vertical layers with varying thickness extending from the surface to 50 hPa and an approximately 10 m midpoint for the lowest (surface) model layer. The simulation time period covers the months from April to September 2011 with a 10 d spin-up period in March.

Emission input data were based on the 2011 National Emissions Inventory (https://www.epa.gov/air-emissions-inventories, last access: 2 October 2019). The raw emission files were processed using version 3.6.5 of the Sparse Matrix Operator Kernel Emissions (SMOKE; https://www.cmascenter.org/smoke/, last access: 2 October 2019) processor to create gridded speciated hourly model-ready input emission fields for input to CMAQ. Electric generating unit (EGU) emissions were obtained using data from EGUs equipped with a continuous emission monitoring system (CEMS). Plume rise for point and fire sources were calculated in-line for all simulations (Foley et al., 2010). Biogenic emissions were generated in-line in CMAQ using BEIS versions 3.61 (Bash et al., 2016). All the simulations employed the bidirectional (bi-di) ammonia flux option for estimating the air-surface exchange of ammonia.

There are four CMAQ simulation scenarios for this study: (1) simulation without LNO (Base), (2) simulation with LNO generated by the scheme based on monthly information from the NLDN (mNLDN), (3) simulation with LNO generated by scheme based on hourly information from the NLDN (hNLDN), and (4) simulation with LNO generated by the scheme parameterizing lightning emissions based on modeled convective activity (pNLDN) as described in detail in Kang et al. (2019). All other model inputs, parameters and settings were the same across the four simulations. The vertical distribution algorithm is the same for all the LNO schemes as also described in Kang et al. (2019).

### 2.3 Observations and analysis techniques

**Figure 1.**
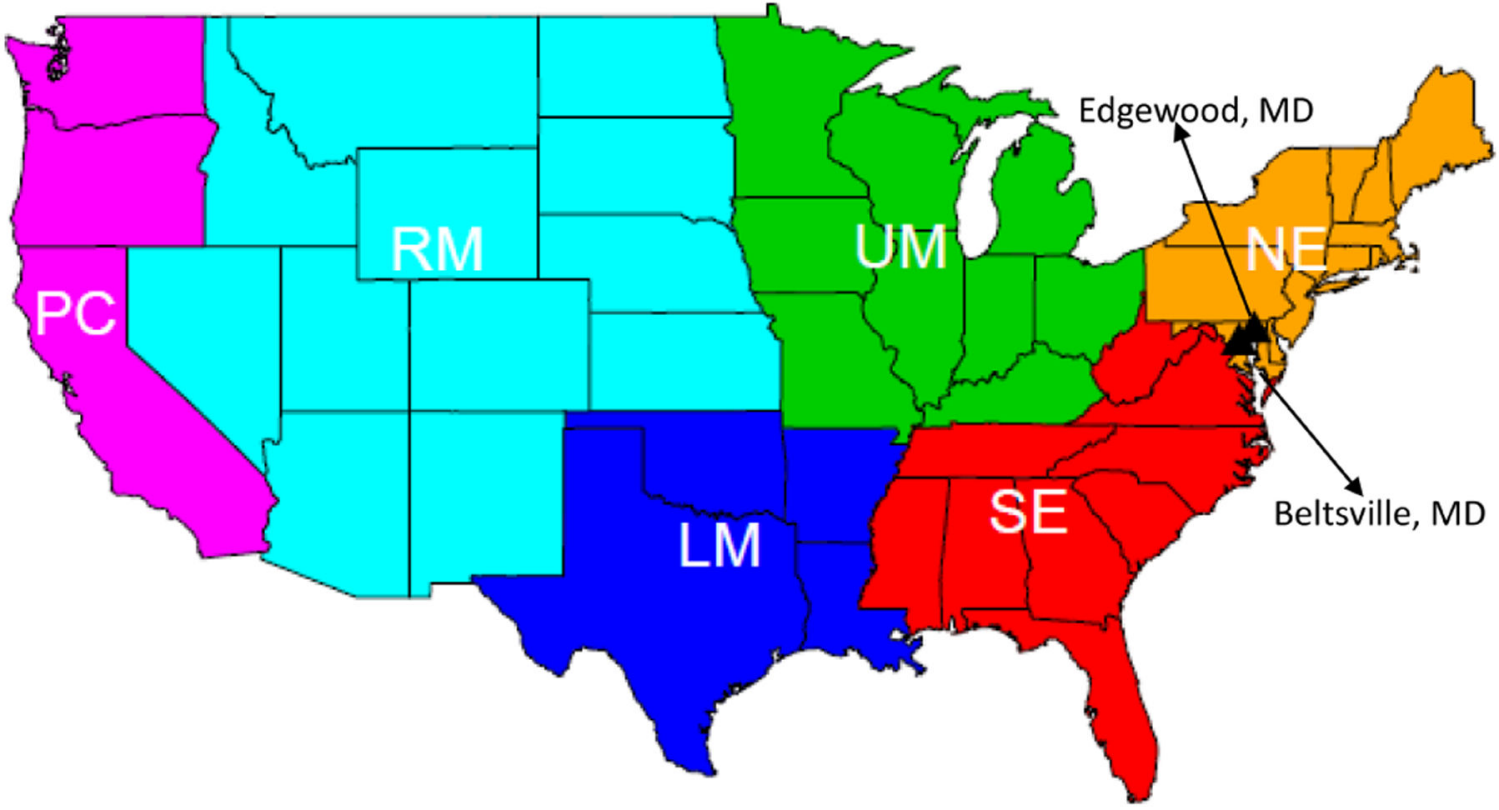
Analysis regions and ozonesonde locations during the 2011 DISCOVER-AQ field study.

To assess the impact of LNO on ground-level air quality, output from the various CMAQ simulations were paired in space and time with observed data from the U.S. EPA Air Quality System (AQS; https://www.epa.gov/aqs, last access: 2 October 2019) for hourly O_3_ and NO_*x*_. To evaluate the vertical distribution, measurements of trace species from the Deriving Information on Surface Conditions from Column and Vertically Resolved Observations Relevant to Air Quality (DISCOVER-AQ; http://www.nasa.gov/mission_pages/discover-aq, last access: 2 October 2019) campaign conducted in the Baltimore–Washington region (e.g., Crawford and Pickering, 2014; Anderson et al., 2014; Follette-Cook et al., 2015) were used. During this campaign, the NASA P-3B aircraft measured trace gases including O_3_, NO, and NO_2_. Vertical profiles were obtained over seven locations – Beltsville (Be), Padonia (Pa), Fair Hill (Fa), Aldino (Al), Edgewood (Ed), Essex (Es), and Chesapeake Bay (Cb) from approximately 0.3 to 5 km above ground level during P-3B flights over 14 d in July 2011. During this same period, ozonesonde measurements were taken that extended from ground level through the entire model column at two locations (Beltsville, MD, and Edgewood, MD, as shown in Fig. 1). Inclusion of LNO estimates in the CTM simulations also has an important impact on model estimated wet deposition of nitrate. Therefore, assessment was also performed using data from the National Atmospheric Deposition Program’s National Trends Network (NADP NTN, http://nadp.slh.wisc.edu/ntn, last access: 2 October 2019).

Since lightning activity and LNO exhibit distinct spatial variations (Kang and Pickering, 2018), analysis was conducted for the model domain over the contiguous United States and then for each region as shown in Fig. 1. Emphasis is placed on two regions, the southeast (SE) and the Rocky Mountains (RM), where lightning activity is more prevalent and LNO has the greatest impact on model predictions as shown in the Results section – increasing model bias in the SE and decreasing bias in the RM. The commonly used statistical metrics, root mean square error (RMSE), normalized mean error (NME), mean bias (MB), normalized mean bias (NMB), and correlation coefficient (*R*) in the model evaluation field, as defined in Kang et al. (2005) and Eder et al. (2006), were calculated to assess the basic performance differences among all the model cases for their ground-level air quality predictions.

## 3 Results

### 3.1 Ground-level evaluation for O_3_ and NO_*x*_

#### 3.1.1 Statistical performance metrics

Tables 1 and 2 display the statistical model performance metrics for daily maximum 8 h (DM8HR) O_3_ and daily mean NO_*x*_ mixing ratios over the domain and each analysis region for all four model cases in July 2011 (Base, mNLDN, hNLDN, and pNLDN). The best performance metrics among the model cases are highlighted in bold. As shown in Table 1, for DM8HR O_3_, the Base simulation has the lowest MB and NMB values over the domain, while hNLDN produced the smallest RMSE and NME values. The mNLDN generated the largest values for both error (RMSE and NME) and biases (MB and NMB), followed by pNLDN, and all model cases with LNO exhibit slightly higher correlation coefficients than the Base simulation. Additionally, the hNLDN simulation exhibited higher correlation and lower bias and error relative to the measurements indicating the value of higher-temporal-resolution lightning activity for representing the associated NO_*x*_ emissions and their impacts on tropospheric chemistry.

Examining the regional results for DM8HR O_3_ in Table 1, the statistical measures indicate that in the northeast (NE), hNLDN outperformed all other model cases with the lowest errors and biases and highest correlation coefficient. In the southeast (SE), the Base simulation performed better with the lowest errors and mean biases, but the correlation coefficient (*R*) value for hNLDN is slightly higher. Among all the LNO cases, mNLDN produced the worst statistics in this region. Historically, CTMs tend to significantly overestimate surface O_3_ in the southeast US (Lin et al., 2008; Fiore et al., 2009; Brown-Steiner et al., 2015; Canty et al., 2015), and this is partially driven by a likely overestimation of anthropogenic NO_*x*_ emissions (Anderson et al., 2014). Thus, even though lightning is known to impact ambient air quality, including this additional NO_*x*_ source can worsen biases in model O_3_ in some locations and time periods due to other errors in the modeling system. As noted in Table 1, compared to the Base, the MB values in the SE increased by about 1.6 ppb with mNLDN and increased by less than 1 ppb with hNLDN and pNLDN. Nevertheless, the correlation coefficients for mNLDN and pNLDN were almost the same with the Base, and hNLDN was slightly higher (0.77 compared to 0.76). These correlations indicate that even though additional NO_*x*_ increases the mean bias, when it is added correctly in time and space, as with the case of hNLDN, the spatial and temporal correlation are slightly improved. In the Upper Midwest (UM), the lowest errors and biases among the model cases are associated with hNLDN, while the worst performance is with mNLDN. In the Lower Midwest (LM), hNLDN performed comparable with the Base, with hNLDN having the highest correlation and lowest mean errors, while the Base has the lowest mean biases. The Rocky Mountain (RM) region is the only region that shows an underestimation of DM8HR O_3_. In this region all the model cases with LNO outperformed the Base case in all the metrics. Among the three model cases with LNO, mNLDN produced the lowest MB and NMB values, while hNLDN had the lowest RMSE and NME, and the highest correlation. In the Pacific Coast (PC) region, lightning activity is generally very low compared to other regions (Kang and Pickering, 2018). All model cases with LNO outperformed the Base case, especially hNLDN which had the lowest mean error and bias and highest correlation among all the cases.

Most of the NO_*x*_ produced by lightning is distributed in the middle and upper troposphere with only a small portion being distributed close to the surface. As a result, the impact on ground-level NO_*x*_ mixing ratios is small. Table 2 shows all the model cases produced similar statistics for the daily mean NO_*x*_ mixing ratios at AQS sites across the domain and within all the subregions. Although the changes in model performance are small, the model cases with LNO exhibit similar or slightly better performance than the Base case.

#### 3.1.2 Time series

**Figure 2.**
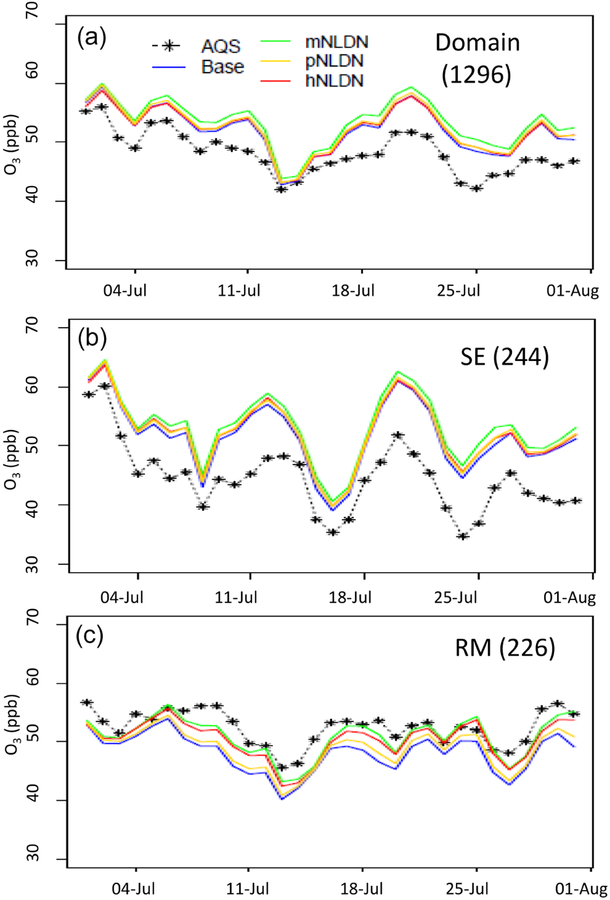
Time series of regional-mean daily maximum 8 h O_3_ comparing observations (AQS) and CMAQ model predictions using the LNO_*x*_ schemes to Base simulation for the domain (a), for SE (b), and for RM (c) in July 2011. The numbers in the parentheses following the region names are the number of AQS sites.

Figure 2 presents time series of regional-mean observed and modeled DM8HR O_3_ for the entire domain and the SE and RM regions during July 2011. Over the domain and in SE, all the model cases overestimate the mean DM8HR O_3_ mixing ratios on all days with the Base being the closest to the observations. The hNLDN is almost the same as the Base with slightly higher values on some days. Among all the cases, mNLDN produced the highest values on almost all days through the month, on the order of 1–2 ppb higher than the Base. In contrast, in the RM region, the Base significantly underestimates DM8HR O_3_ mixing ratios on all the days during the month, while all model cases with LNO improved model predictions relative to observations in the region. Among the three model cases with LNO, mNLDN produced the lowest bias for all the days, closely followed by hNLDN.

**Figure 3.**
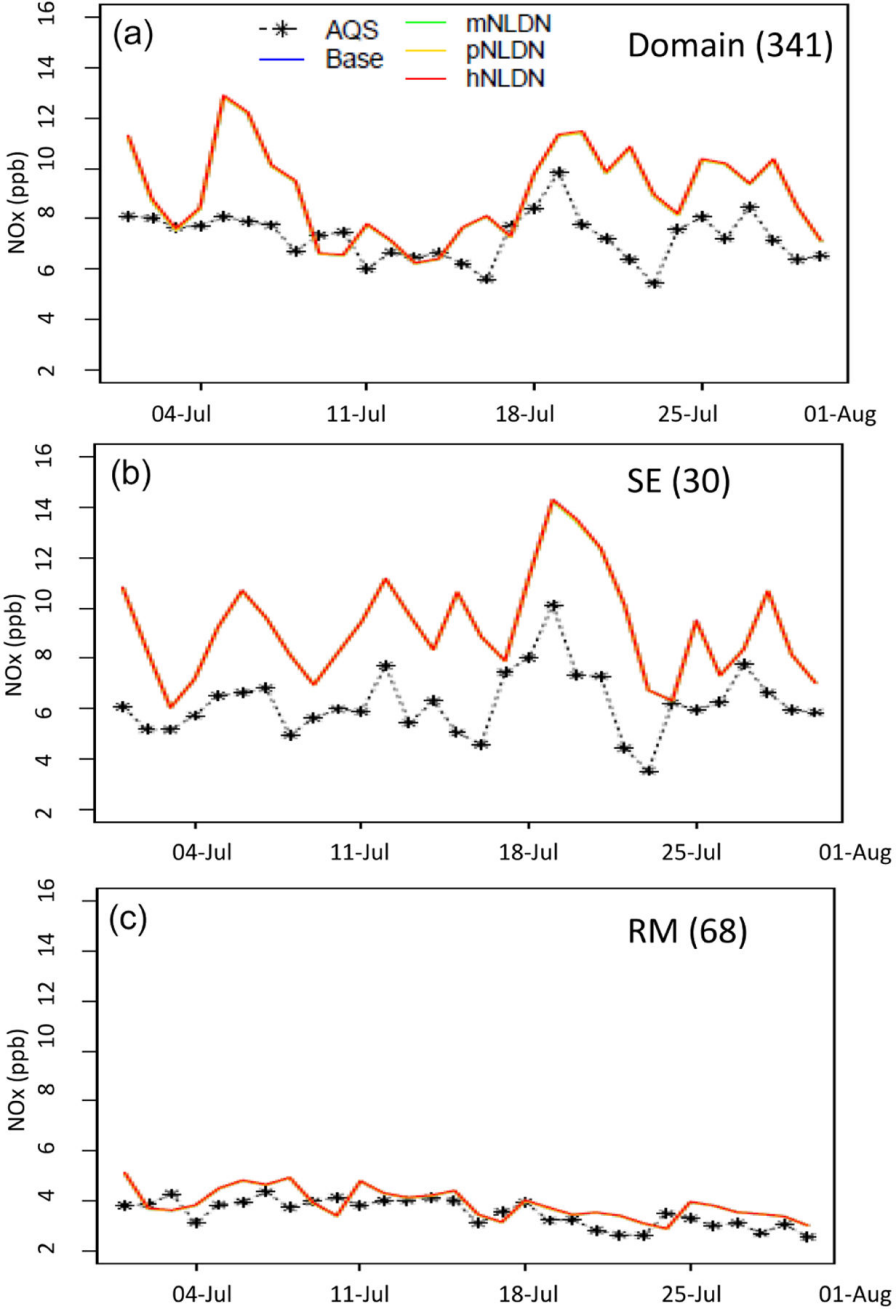
Time series of daily mean NO_*x*_ over the domain (a), SE (b), and RM (c) in July 2011. The numbers in the parentheses following the region names are the number of AQS sites.

Figure 3 displays the average daily mean NO_*x*_ mixing ratios at AQS sites over the same regions as in Fig. 2. On most of the days in July 2011, over the domain and in the SE, the model overestimate NO_*x*_ values, and on almost half of the days the overestimation is significant (up to 100 %). As noted in Table 2, on average, the overestimation is ~ 17 % over the domain and ~ 43 % in SE. However in RM, the predicted NO_*x*_ mixing ratios closely follow the daily observations and on average the modeled and observed magnitude is almost identical (~ 3 % difference). All the model cases, with or without LNO, produced almost the same mean NO_*x*_ mixing ratios at the surface. However, the different cases produce different levels of LNO in the middle and upper troposphere, resulting in differences in O_3_ production and transport which impact radiative forcing and also downwind ground-level O_3_ levels. We further explore these features in Sect. 3.2 which presents evaluation of modeled vertical pollutant distributions.

#### 3.1.3 Diurnal variations

**Figure 4.**
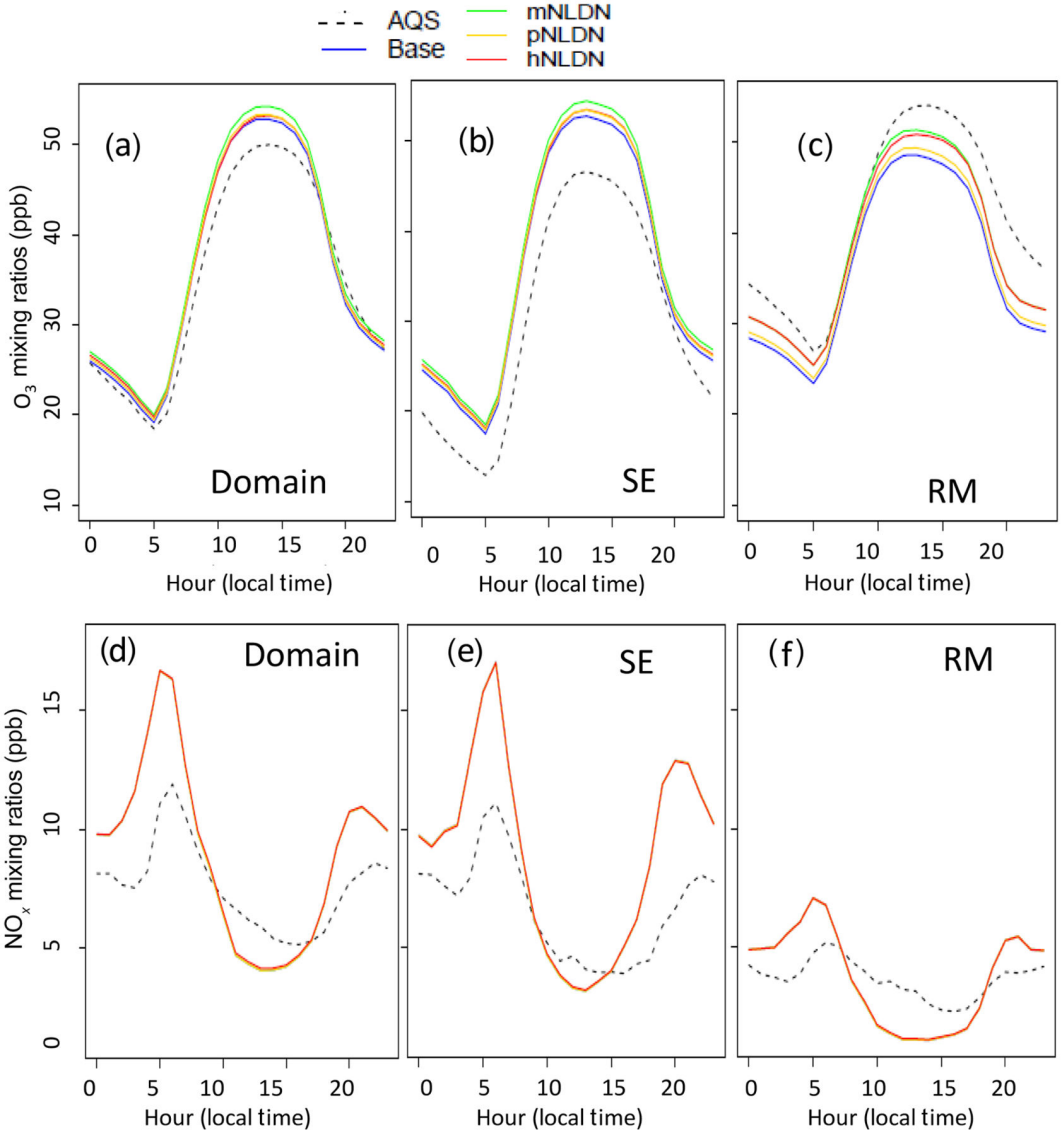
Diurnal profiles for hourly O_3_ and NO_*x*_ over the domain (**a**, **d**), SE (**b**, **e**), and RM (**c**, **f**) in July 2011.

Diurnal plots are used to further examine differences in model evaluation for O_3_ and NO_*x*_. Figure 4 shows the mean diurnal profiles for hourly O_3_ and NO_*x*_ over the entire domain, SE, and RM. On a domain mean basis, all model cases overestimate O_3_ during the daytime hours, while in the SE the overestimation spans all the hours. In RM, the model cases significantly underestimate O_3_ across all the hours except for a few early morning hours, when the model predicted values are very close to the observations. Among all the model cases, as expected, the most prominent differences occurred during the midday hours when the photochemistry is most active. However, the difference between hNLDN (and mNLDN) and the Base is also significant during the night in the RM region, even though the O_3_ levels are low. This may be attributed to NO_*x*_-related nighttime chemistry in part caused by freshly released NO by cloud-to-ground lightning flashes. The diurnal variations of NO_*x*_ are similar over the domain and in the regions for all model cases. Appel et al. (2017) reported a significant overestimation of NO_*x*_ mixing ratios at AQS sites during nighttime hours and underestimation during daytime hours. The bias pattern is identical for all of the LNO model cases evaluated here (Fig. 4).

#### 3.1.4 Spatial variations

**Figure 5.**
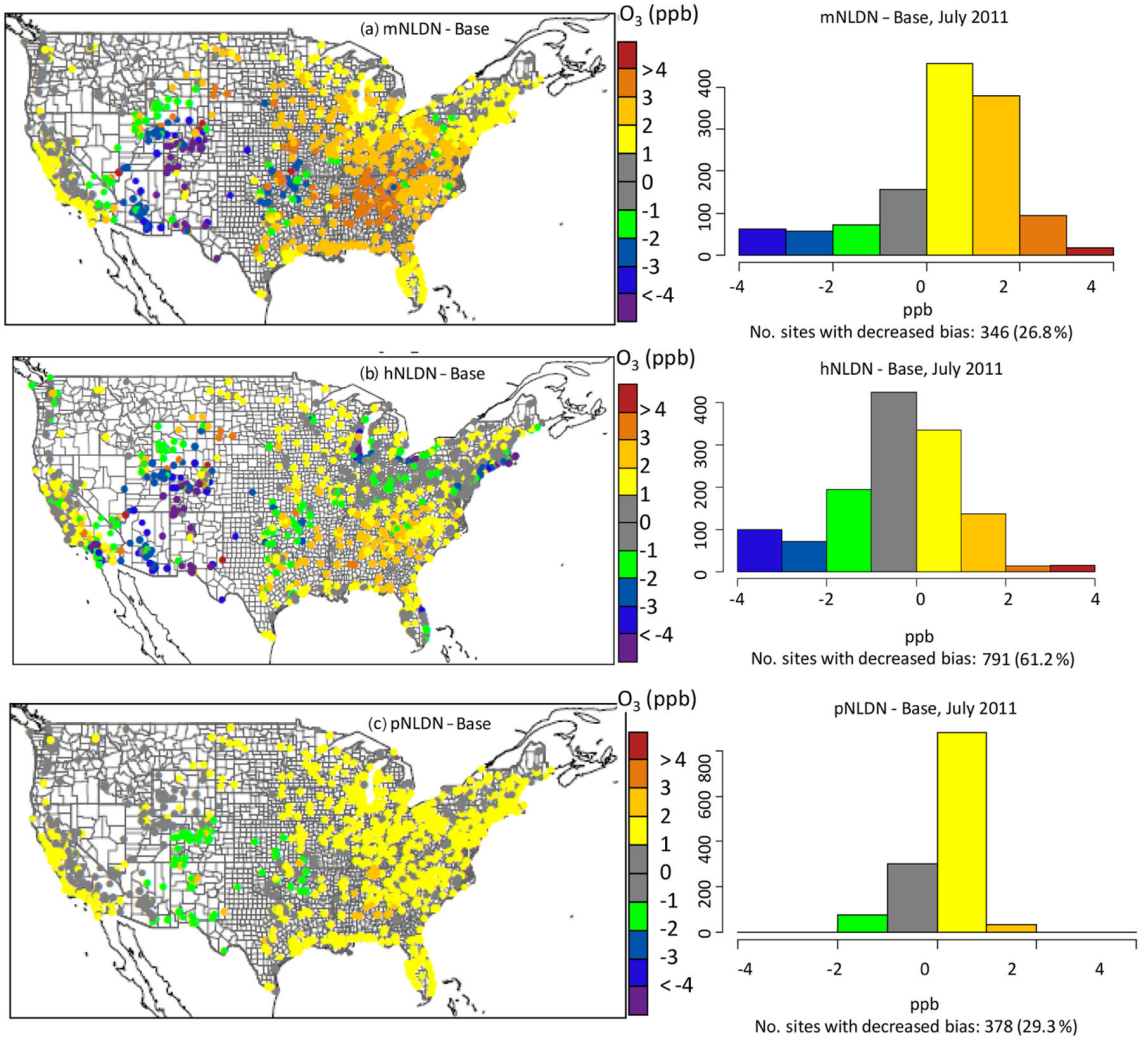
Spatial maps of the mean bias of DM8HR O_3_ (model – observation) differences between model case with LNO_*x*_ and the Base as well as the corresponding histograms indicating the number of sites with decreased mean bias for each pair of model cases in July 2011.

Figure 5 shows the impact of the different LNO schemes on model performance for DM8HR O_3_ at AQS sites. The spatial maps show the difference in absolute MB between the cases with lightning NO_*x*_ emissions and the Base and is calculated as follows. First, the absolute MB was calculated at each site for each case, e.g., |MB_[Base–Obs]_|, then the difference in absolute MB was calculated between model cases, e.g., |MB_[hNLDN–Obs]_|− |MB_[Base–Obs]_|. The histograms of the diffrences in absolute MB between model cases in fig. 5 are absolute provided to show the distribution of the change in model performance across space, i.e., the frequency of an improvement in model performance versus a degradation in model performance between cases. As shown in Fig. 5, the mNLDN shows increased model bias in the east US and along the California coast, but reduced model bias in the RM. At a majority of the AQS sites, it increases the model bias (only decreases at 26.8 % (346) of the sites). The hNLDN also significantly reduces model bias in the RM with a moderate increase in the SE. Overall, in the hNLDN, the mean bias decreased at 61.2 % (791) of AQS sites. Similar to mNLDN, increases in mean bias are noted at 29.3 % (378) of the AQS sites in the pNLDN simulation. As noted in the histograms, the distribution of the model bias in the pNLDN is much narrower than both mNLDN and hNLDN, eliminating the large bias increases in mNLDN and the significant bias decreases in hNLDN.

### 3.2 Vertical evaluation for O_3_ and NO_*x*_

#### 3.2.1 Ozone-sonde observations

**Figure 6.**
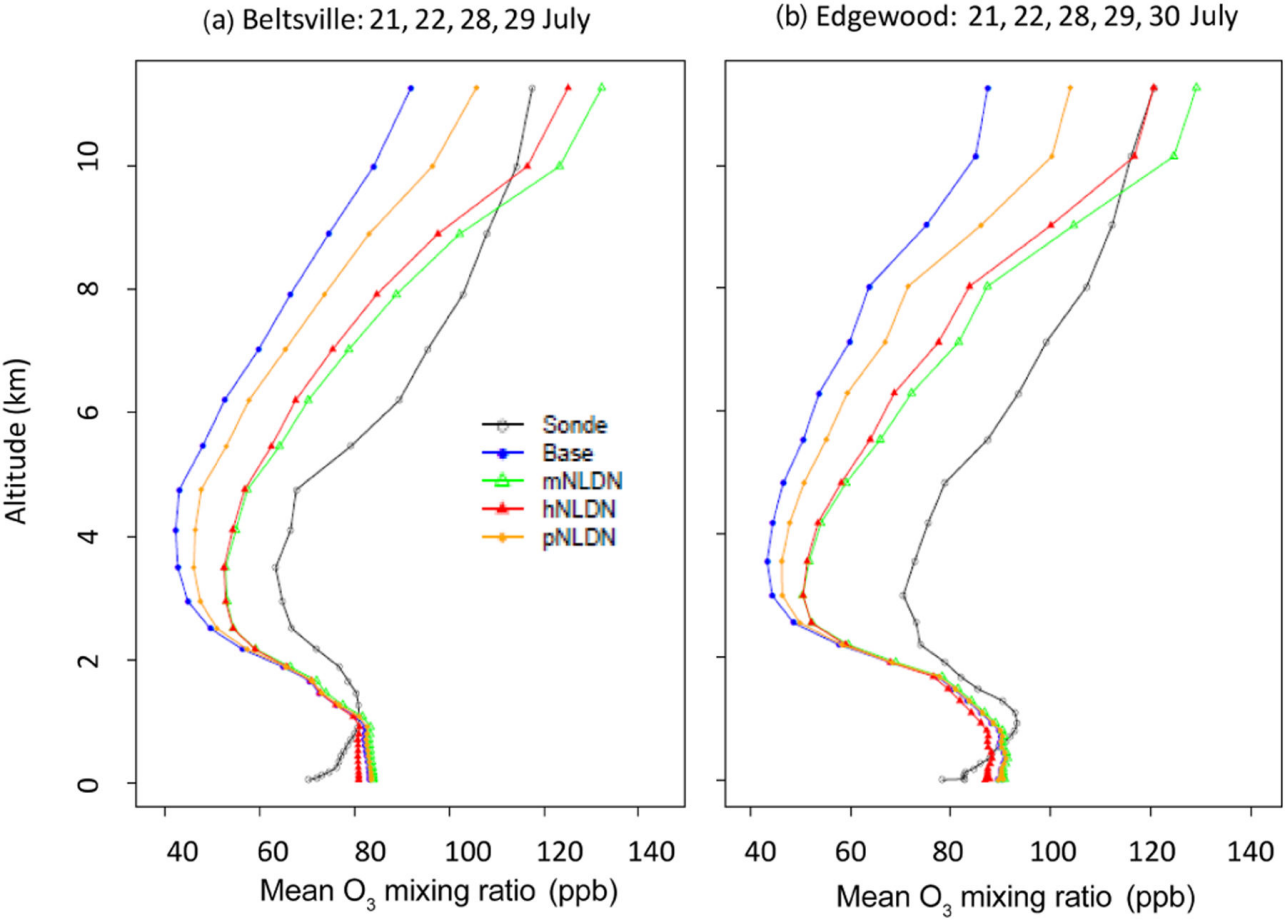
Vertical profiles of O_3_ mixing ratios from ozonesonde measurements and model simulations at Beltsville, MD (**a**); and Edgewood, MD, (**b**) on the days when lightning NO produced significant impact on O_3_ during the DISCOVER-AQ field study in July 2011.

A large source of uncertainty in the specification of LNO is its vertical allocation, which can impact the model’s ability to accurately represent the variability in both chemistry and transport. To further assess the impact of the vertical LNO specification on model results, we compared vertical profiles of simulated model O_3_ with extensive ozonesonde measurements available during the study period. Figure 6 presents the vertical profiles for O_3_ sonde measurements and paired model estimates of all model cases at Beltsville, MD, and Edgewood, MD. At each location, observations from multiple days are available (one or two soundings per day) during the 2011 DISCOVER-AQ campaign in July 2011. The model evaluation was limited to days where the inclusion of LNO has an obvious impact (the mean vertical profiles of LNO cases are separable from that of the Base case) on the model estimates (21, 22, 28, and 29 July at Beltsville, and 21, 22, 28, 29, and 30 July at Edgewood). We paired the observed data with model estimates in time and space and averaged the model and observed values at each model layer. Only data below 12 km altitude are plotted in Fig. 6 to exclude possible influence of stratospheric air on O_3_. As can be seen in Fig. 6, at both locations the Base case underestimates O_3_ mixing ratios above about 1 km, but overestimates values closer to the surface. When LNO is included in the simulations, the predicted O_3_ mixing ratios increase relative to the Base case starting around 2 km, with greater divergence from the Base case at higher altitudes. The two model cases, hNLDN and mNLDN, produced similar O_3_ levels from the surface to about 6 km, but above that altitude the mNLDN ozone mixing ratios were higher. All the model cases with LNO performed much better aloft than the Base case. Near the surface, all the model cases overestimated O_3_, however hNLDN had smaller bias than the other simulations. This may be attributed to the fact that only hNLDN used the observed lightning flash data directly, and as a result, LNO was estimated more accurately in time and space. This improvement in model bias at the surface is further investigated in the next section using evaluation against P-3B measurements.

#### 3.2.2 P-3B measurement

**Figure 7.**
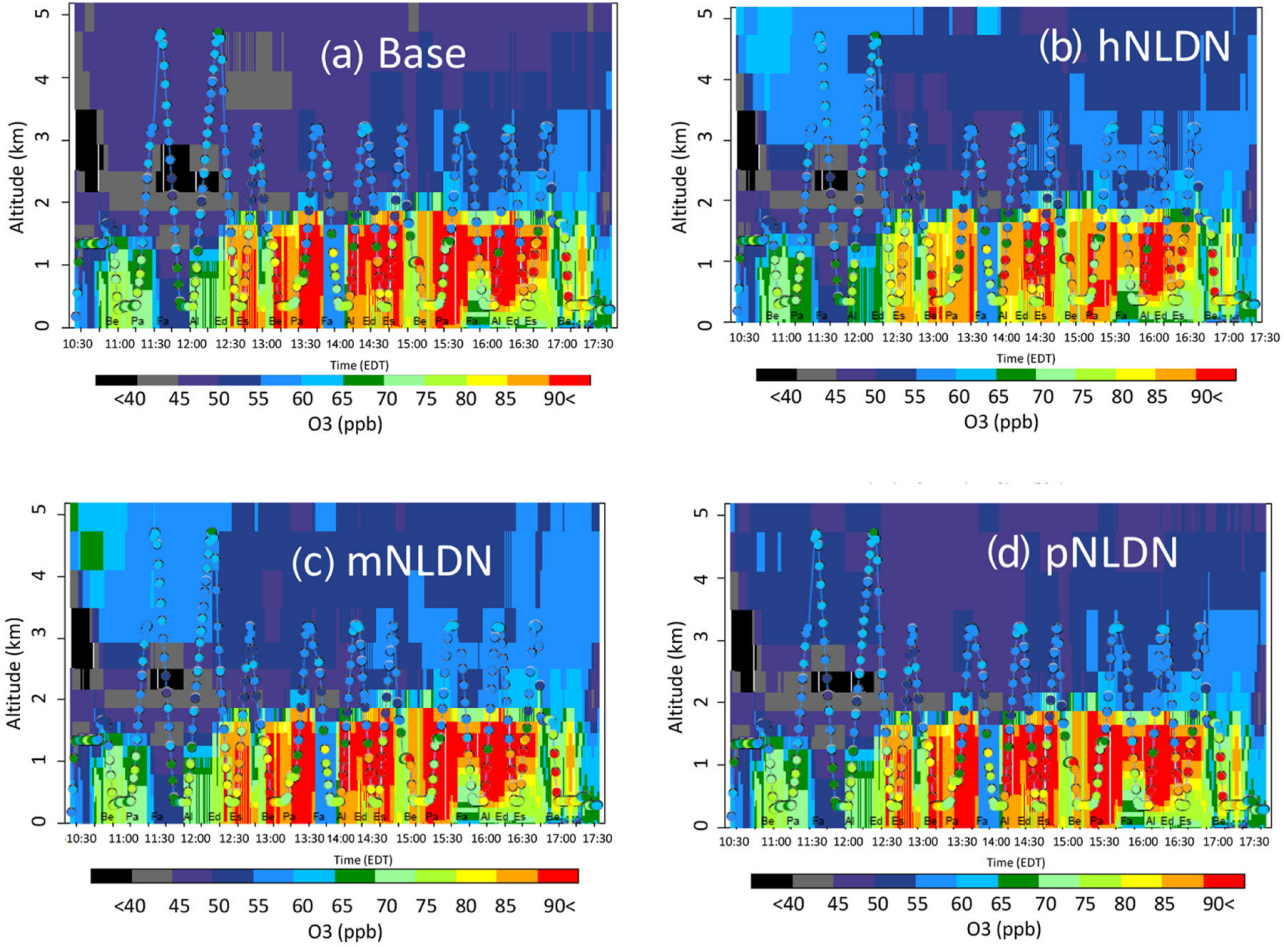
Overlay of P-3B-observed O_3_ (1 min mean values) over the corresponding vertical cross sections of simulated values extracted at the flying locations on 28 July 2018, (**a**) Base, (**b**) hNLDN (**c**) mNLDN, and (**d**) pNLDN. The letters marked at the bottom of the plots are P-3B spiral sites, Be: Beltsville, Pa: Padonia, Fa: Fair Hill, Al: Aldino, Ed: Edgewood, and Es: Essex.

Extensive measurements of lower tropospheric chemical composition distributions over the northeastern US are available from instruments onboard the P-3B aircraft on 14 d of the DISCOVER-AQ campaign. We utilize measurements from one of the days (28 July 2011) with noticeable (the mean vertical profiles of LNO cases are separable from that of the Base case) lightning impacts, to evaluate the model simulations. Figure 7 shows measured O_3_ mixing ratios overlaid on the modeled vertical time section for 10:30–17:30 UTC. The color-filled circles represent measured O_3_ mixing ratios averaged over 60 s and the background is the model estimated vertical profiles from the grid cells containing the P-3B flight path for that hour and location. As indicated in the Base case (Fig. 7a), the model tends to overestimate O_3_ mixing ratios from the surface to about 2 km, but it tends to underestimate at altitudes above 2 km. The hNLDN reduced the overestimation below 2 km, e.g., fewer grid cells with mixing ratios above 90 ppb (shown in red). The other two cases (mNLDN and pNLDN) did not produce the same improvement near the surface. The hNLDN also decreases the underestimation aloft compared to the Base case with O_3_ mixing ratios in the 55–65 ppb range (light blue colors), better matching the measured values. This decrease in underestimation aloft is also seen in the mNLDN case, but to a lesser degree while the pNLDN case shows only slight improvement aloft over the Base simulation.

**Figure 8.**
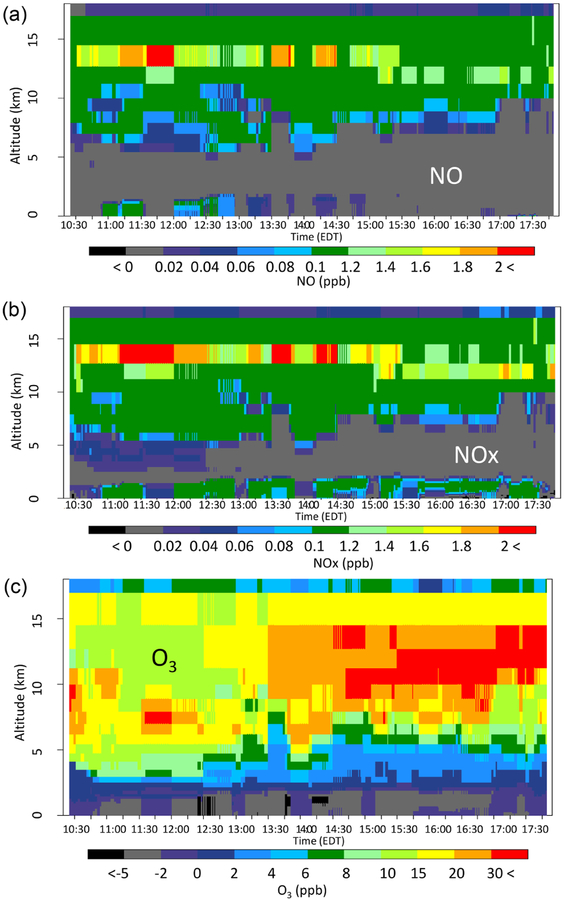
The vertical-time difference between hNLDN and Base during the P-3B flight period on 28 July 2011 for (**a**) NO, (**b**) NO_*x*_, and (**c**) O_3_.

**Figure 9.**
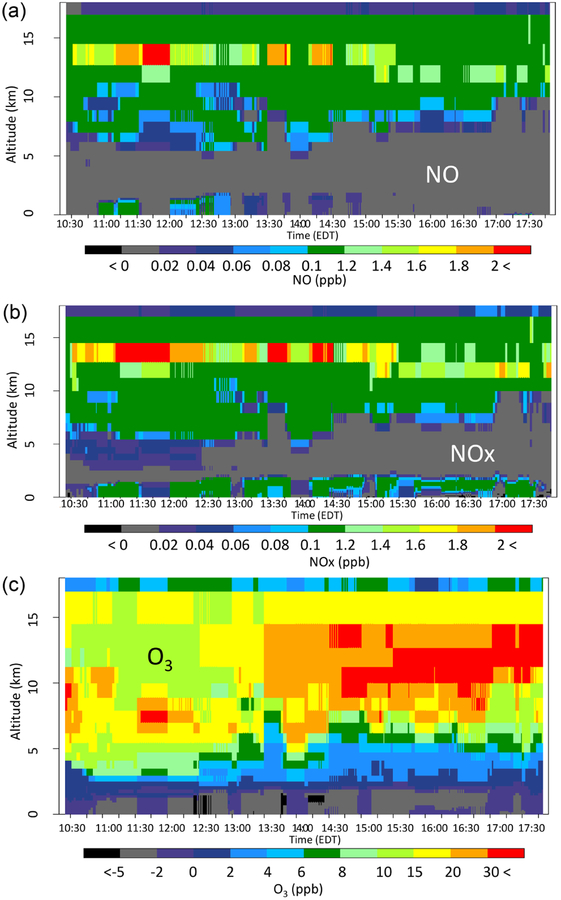
The vertical-time difference between mNLDN and Base during the P-3B flight period on 28 July 2011 for (**a**) NO, (**b**) NO_*x*_, and (**c**) O_3_.

**Figure 10.**
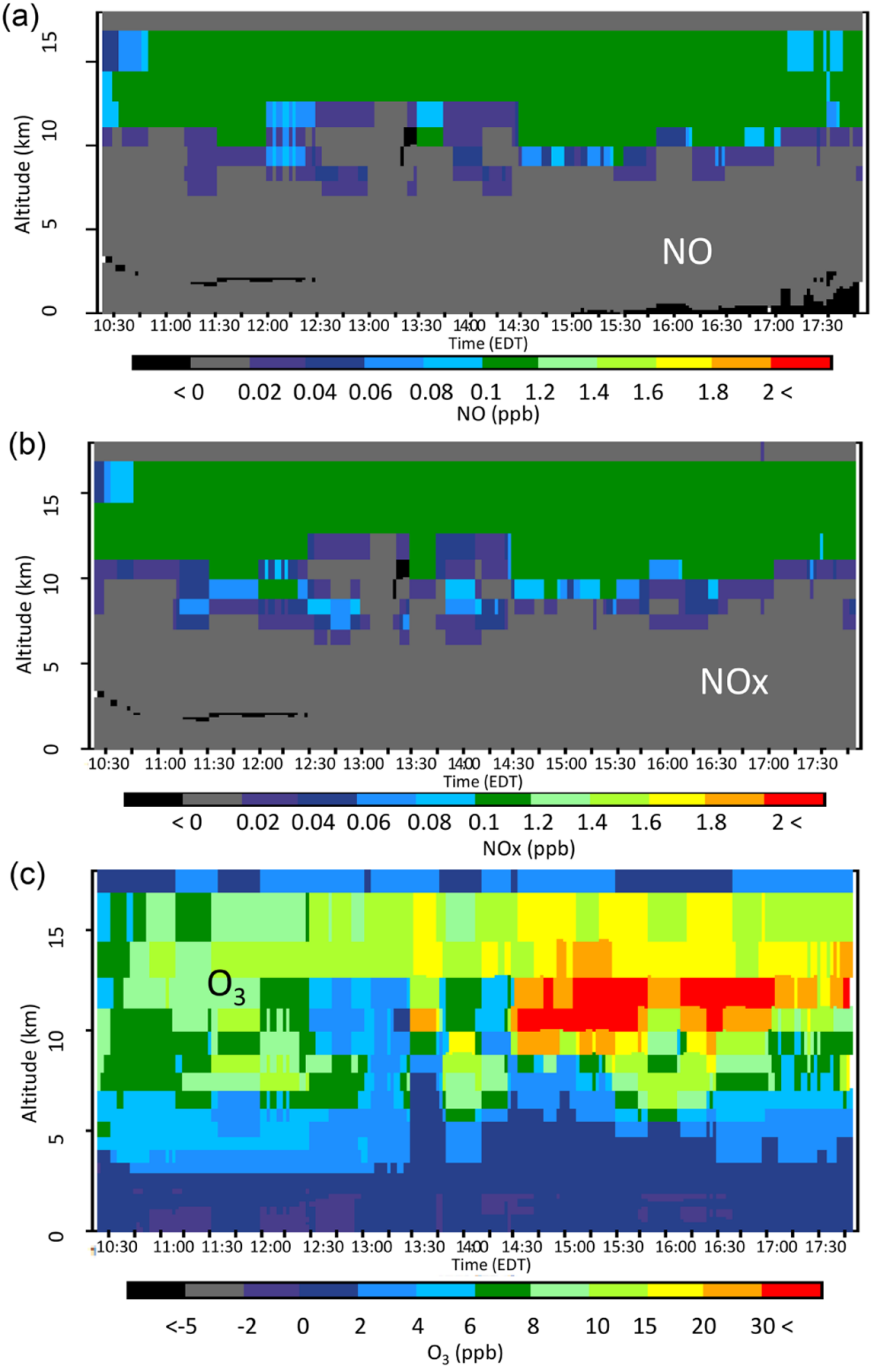
The vertical-time difference between pNLDN and Base during the P-3B flight period on 28 July 2011 for (**a**) NO, (**b**) NO_*x*_, and (**c**) O_3_.

To further differentiate the three LNO model cases, Figs. 8–10 show the difference in the time sections between each of the model cases with LNO and the Base for NO, NO_*x*_, and O_3_ from all the model layers along the P-3B flight path on 28 July. As seen in Fig. 8, the hNLDN scheme injected most NO above 5 km with a peak between 13 and 14 km and only a small amount near the surface. After release into the atmosphere, NO is quickly converted into NO_2_ in the presence of O_3_, and these collectively result in the NO_*x*_ vertical time section (local production plus transport) shown in the middle panel of Fig. 8. NO_*x*_ is further mixed down through the time section and is more persistent along the flight path near the surface than NO is. As a result, significant O_3_ is produced above 3 km, and the maximum O_3_ difference appears between 9 and 14 km during the early afternoon hours (from 13:30 to 17:30 Eastern Daylight Time). However, from surface to about 2 km, O_3_ is reduced consistently across the entire period, and this is the result of O_3_ titration by NO from cloud-to-ground lightning flashes that must have been transported to this layer by storm down-drafts. Since O_3_ is significantly underestimated above 3 km and overestimated near the surface by the Base model, the inclusion of LNO greatly improved the model’s performance under both conditions.

Comparison of Fig. 9 (mNLDN) with Fig. 8 (hNLDN) reveals that the time sections of NO and NO_*x*_ are similar above 5 km but dramatically different near the surface. The near-surface increase in ambient NO noted in the hNLDN is absent in mNLDN, and in fact there are some small decreases in NO, although the reason for this is unclear. The increase in O_3_ aloft in the mNLDN case is similar to that seen in the hNLDN case. However, the near-surface reduction in O_3_ is almost absent. In the pNLDN case (Fig. 10), NO mixing ratios are much less than those in hNLDN and mNLDN in the upper layers as a result of less column NO being generated by the linear parameterization. The resulting NO_*x*_ time section is also smoothed. The pNLDN time sections for NO, NO_*x*_, and O_3_ near the surface are similar to the mNLDN case with no change or small decreases compared to the Base case. O_3_ mixing ratios increase by more than 30 ppb during the afternoon hours between 10 and 13 km in the pNLDN case, however the increase is not as intense and widespread as the other cases. In summary, the hNLDN scheme produces estimates that are more consistent with measurements at the surface and aloft, compared to the other simulations, reflecting the advantage of using the spatially and temporally resolved observed lightning flash data. The model performance improvement for simulated O_3_ distributions also suggests robustness in the vertical distribution scheme when LNO is generated at the right time and location.

To corroborate the above time section distributions of NO, NO_*x*_, and O_3_ in the lightning cases, the lightning NO emissions are traced back to 28 July for each case. It is found that in all cases, the lightning NO was injected approximately 200 km upwind (northwest) of the flight path. The hNLDN case captured two injections: one occurred during the morning hours (05:00 to 07:00 EDT) and the other happened during the afternoon hours (after 02:30 EDT). Both mNLDN and pNLDN captured the afternoon lightning event at the later time (after 03:30 EDT for mNLDN and after 04:30 for pNLDN) with varying intensity, but neither captured the morning lightning event, which explains why the increase in NO and NO_*x*_ in the hNLDN case (Fig. 8) did not occur in the mNLDN and pNLDN cases (Figs. 9 and 10). Also note that the significant increase of NO during the time period from 11:00 to 13:00 EDT occurred about 5 h after the lightning NO was injected at about 200 km upwind in the hNLDN case.

**Figure 11.**
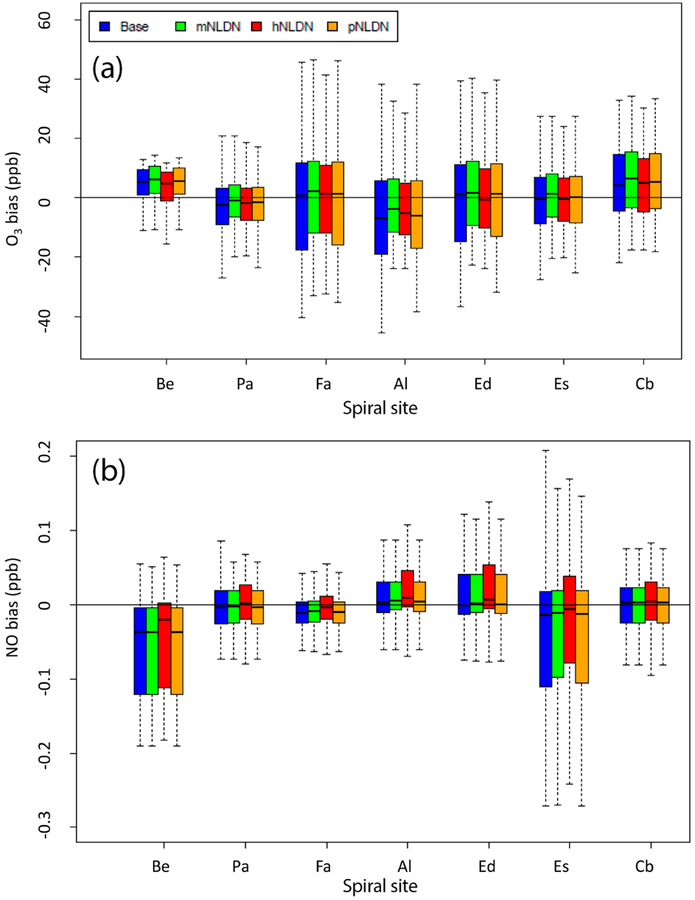
Bias (model – observation) distributions of O_3_ (**a**) and NO (**b**) at each P-3B spiral site on 21, 22, 28, and 29 July 2011. Be: Beltsville, Pa: Padonia, Fa: Fair Hill, Al: Aldino, Ed: Edgewood, Es: Essex, and Cb: Chesapeake Bay.

To expand on the evaluation in Figs. 7–10 which focused on measurements from 28 July 2011, we retrieved all the P-3B measurements on days with noticeable lightning impact (21, 22, 28, and 29 July). The 3-D paired observation–model data were grouped together by spiral site and the mean biases (model – observation) were plotted in Fig. 11 (a and b) for O_3_ and NO, respectively. The boxplots for O_3_ in Fig. 11a suggests that the Base exhibited larger bias with greater spread (i.e., larger interquartile range) than other model cases incorporating LNO at most of the locations where aircraft spirals were conducted. At all locations except Aldino, the lowest mean biases in simulated NO and O_3_ are noted in the hNLDN simulation.

### 3.3 Deposition evaluation for nitrate

**Figure 12.**
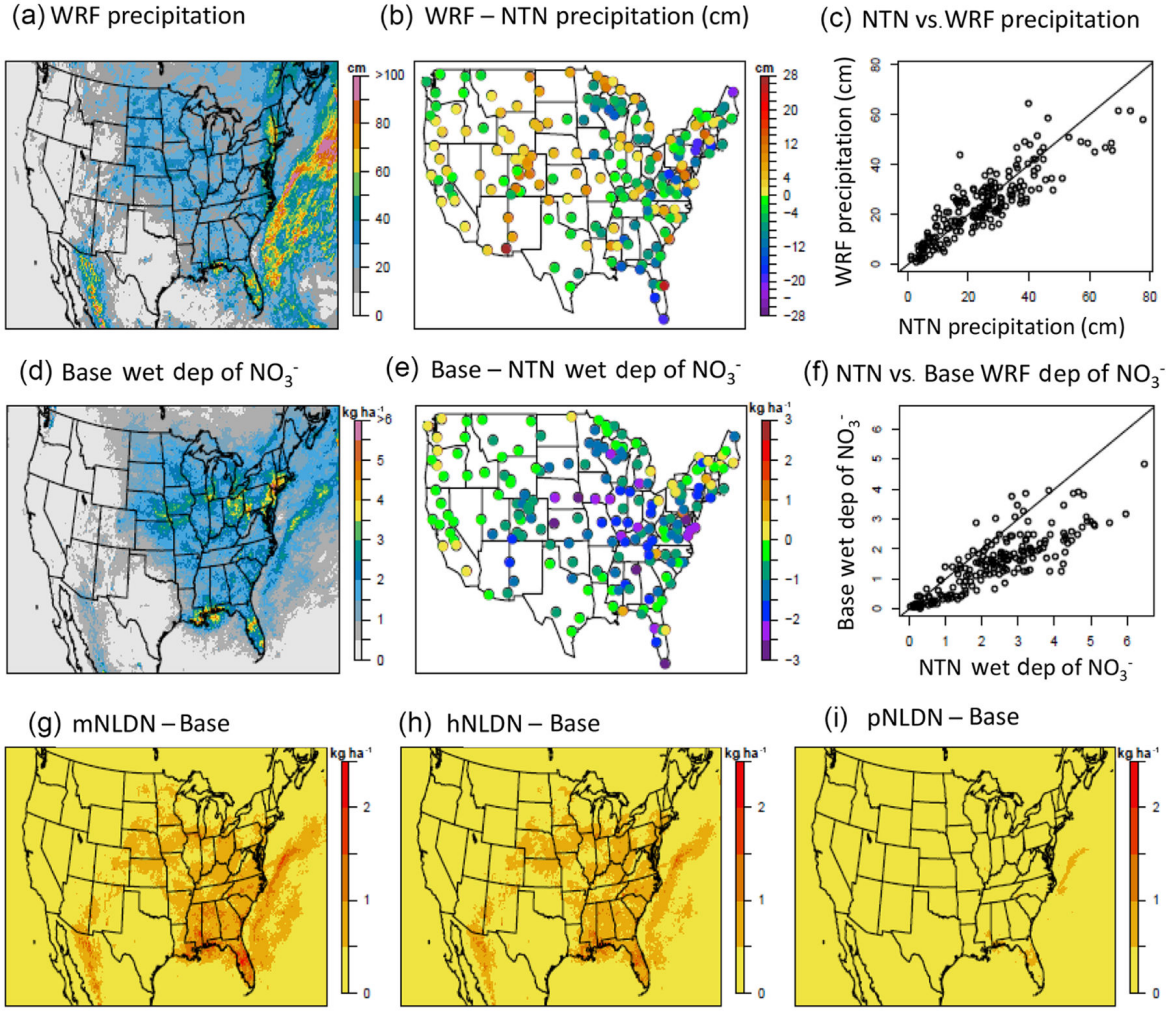
(**a**)–(**c**) shows precipitation estimates from WRF (**a**), the bias in the WRF predicted precipitation at NTN locations (**b**), and the corresponding scatter plots (**c**). (**d**)–(**f**) shows wet deposition (dep) of nitrate estimates from the Base simulation (**d**), the bias in the Base model estimates of wet deposition of ${\text{NO}}_{3}^{-}$ at NADP NTN locations (**e**), and the corresponding scatter plots (**f**). (**g**)–(**i**) shows the difference in the LNO_*x*_ sensitivity simulations and the Base case estimates of wet deposition of ${\text{NO}}_{3}^{-}$ for mNLDN – Base (g); hNLDN – Base (**h**), and pNLDN – Base (**i**). All maps are based on accumulated values (precipitation or wet deposition) during June–August 2011. Precipitation totals are in centimeters (cm) and wet deposition totals are in kilograms per hectare (kg ha^−1^).

In addition to contributing to tropospheric O_3_ formation, NO_*x*_ oxidation also leads to gaseous nitric acid and particulate nitrate which are eventually removed from the atmosphere by dry and wet deposition of nitrate (${\text{NO}}_{3}^{-}$). As a result, inclusion of NO_*x*_ from lightning also plays an important role in nitrogen deposition modeling. To assess the impacts of incorporating LNO emissions on simulated oxidized nitrogen deposition, we compared model estimated amounts of precipitation from the NTN network (http://nadp.slh.wisc.edu/ntn/, last access: 2 October 2019) and wet deposition of ${\text{NO}}_{3}^{-}$ with measurements from the NADP network (http://nadp.slh.wisc.edu/, last access: 2 October 2019). During summer months in 2011 (June–August) the WRF model generally reproduces the observed precipitation with a slight underestimate in the east, but the Base model simulation tends to underestimate wet deposition of ${\text{NO}}_{3}^{-}$ across the domain, with the greatest underestimation in the SE and UM (See Table 3 and Fig. 12). All three LNO simulations increase wet deposition amounts of ${\text{NO}}_{3}^{-}$ and decrease model bias in all regions. The bottom panel of Fig. 12 shows that the mNLDN simulation resulted in the largest increase over the Base model estimates. The NMB is reduced from − 35 % in the Base to −15 % in mNLDN across the domain and from 32 % to −2 % in the SE. The hNLDN shows very similar model performance to the mNLDN case. In contrast, the wet deposition ${\text{NO}}_{3}^{-}$ estimates from the pNLDN case are only slightly higher than the Base case, and as a result the evaluation statistics for pNLDN are very similar to the Base statistics. As discussed earlier, the mNLDN tends to produce the most LNO among the three LNO schemes, thus it results in the smallest errors in terms of wet deposition of ${\text{NO}}_{3}^{-}$ when compared to the Base simulation that significantly underestimated ${\text{NO}}_{3}^{-}$ wet deposition. It should be noted that in addition to the LNO contributions, errors in modeled precipitation amounts and patterns also likely influence the underestimation of ${\text{NO}}_{3}^{-}$ wet deposition.

## 4 Conclusions

A detailed evaluation of lightning NO_*x*_ emission estimation parameterizations available in the CMAQ modeling system was performed through comparisons of model simulation results with surface and aloft air quality measurements.

Our analysis indicates that incorporation of LNO emissions enhanced O_3_ production in the middle and upper troposphere, where O_3_ mixing ratios were often significantly underestimated without the representation of LNO. Though the impact on surface O_3_ varies from region to region and is also dependent on the accuracy of the NO_*x*_ emissions from other sources, the inclusion of LNO, when it is injected at the appropriate time and location, can improve the model estimates. In regions where the Base model estimates of O_3_ were biased high, the inclusion of LNO further increased the model bias, and a systematic increase is noted in the correlation with measurements, suggesting that emissions from other sources likely drive the overestimation. Identifying how errors in emission inputs from different sources interact with errors in meteorological modeling of mixing and transport remains a challenging but critical task. Likewise, all the LNO schemes also enhanced the accumulated wet deposition of ${\text{NO}}_{3}^{-}$ that was significantly underestimated by the Base model without LNO throughout the modeling domain except the Pacific Coast.

Uncertainty remains in modeling the magnitude and spatial, temporal, and vertical distribution of lightning produced NO_*x*_. LNO schemes are built on numerous assumptions and all current schemes also depend on the skill of the upstream meteorological models in describing convective activity. Nevertheless, these schemes reflect our best understanding and knowledge at the time when the schemes were implemented. The use of hourly information on lightning activity yielded LNO emissions that generally improved model performance for ambient O_3_ and NO_*x*_ as well as oxidized nitrogen wet deposition amounts. As more high-quality data from both ground and satellite measurements become available, the performance of the LNO schemes will continue to improve.

Since the pNLDN scheme was developed using historical data correlating lightning activity with convective precipitation, the scheme could be employed for applications involving air quality forecasting and future projections when observed lightning information is not available.

## Figures and Tables

**Table 1.** Statistics of DM8HR O_3_ for all model cases over the domain and analysis regions in July 2011. The best performance metrics among the model cases are highlighted in bold.

Region	Case	Record	OBS (ppb)	MOD (ppb)	RMSE (ppb)	NME (%)	MB (ppb)	NMB (%)	*R*
Domain	Base	36 242	48.21	52.04	12.6	19.2	**3.8**	**8.0**	0.69
	mNLDN	36 242	48.21	53.40	12.9	19.8	5.2	10.8	0.70
	hNLDN	36 242	48.21	52.21	**11.9**	**18.4**	4.0	8.3	**0.72**
	pNLDN	36 242	48.21	52.52	12.7	19.5	4.3	8.9	0.70
NE	Base	5512	50.97	55.08	13.0	17.8	4.1	8.1	0.74
	mNLDN	5512	50.97	55.77	13.4	18.5	4.8	9.4	0.74
	hNLDN	5512	50.97	54.23	**11.9**	**16.7**	**3.3**	**6.4**	**0.75**
	pNLDN	5512	50.97	55.32	13.1	18.0	4.4	8.5	0.74
SE	Base	7061	44.55	51.71	**12.6**	**21.0**	**7.2**	**16.1**	0.76
	mNLDN	7061	44.55	53.33	13.6	236	8.8	19.7	0.76
	hNLDN	7061	44.55	52.30	12.6	21.7	7.8	17.4	**0.77**
	pNLDN	7061	44.55	52.39	13.0	22.0	7.8	17.6	0.76
UM	Base	8072	51.60	58.99	13.6	18.8	7.4	14.3	0.64
	mNLDN	8072	51.60	60.14	14.4	20.5	8.5	16.6	0.64
	hNLDN	8072	51.60	58.35	**12.8**	**18.0**	**6.8**	**13.1**	0.64
	pNLDN	8072	51.60	59.42	13.9	19.4	7.8	15.1	0.64
LM	Base	3609	42.15	46.21	12.4	21.5	**4.1**	**9.6**	0.73
	mNLDN	3609	42.15	47.93	12.9	22.3	5.8	13.7	0.74
	hNLDN	3609	42.15	47.12	**12.3**	**21.3**	5.0	11.8	**0.76**
	pNLDN	3609	42.15	46.93	12.6	21.8	4.8	11.3	0.74
RM	Base	6256	52.52	48.13	11.3	17.0	−4.4	−8.4	0.52
	mNLDN	6256	52.52	50.93	10.2	14.7	−**1.6**	−**3.0**	0.56
	hNLDN	6256	52.52	50.35	**9.9**	**14.4**	−2.2	−4.1	**0.57**
	pNLDN	6256	52.52	48.93	10.9	16.2	−3.6	−6.9	0.53
PC	Base	5570	44.72	47.58	11.7	20.1	2.9	6.4	0.80
	mNLDN	5570	44.72	47.73	11.6	20.0	3.0	6.7	0.80
	hNLDN	5570	44.72	46.65	**11.3**	**19.5**	**1.9**	**4.3**	**0.81**
	pNLDN	5570	44.72	47.62	11.6	20.0	2.9	6.5	0.80

**Table 2.** Statistics of daily mean NO_*x*_ for all model cases over the domain and analysis regions in July 2011. The best performance metrics among the model cases are highlighted in bold.

Region	Case	Record	OBS (ppb)	MOD (ppb)	RMSE (ppb)	NME (%)	MB (ppb)	NMB (%)	*R*
Domain	Base	6912	7.58	8.88	**8.7**	62.6	**1.3**	17.1	0.54
	mNLDN	6912	7.58	8.87	**8.7**	**62.5**	**1.3**	**17.1**	0.54
	hNLDN	6912	7.58	8.92	8.7	62.7	1.3	17.7	**0.55**
	pNLDN	6912	7.58	8.87	**8.7**	62.5	**1.3**	17.1	0.54
NE	Base	989	10.48	9.72	**7.0**	46.0	−0.8	−7.3	0.55
	mNLDN	989	10.48	9.71	**7.0**	**46.0**	−0.8	−7.3	**0.55**
	hNLDN	989	10.48	9.77	7.1	46.1	−**0.7**	−**6.8**	0.55
	pNLDN	989	10.48	9.72	**7.0**	46.0	−0.8	−7.3	0.55
SE	Base	645	6.44	9.18	7.2	75.3	2.7	42.6	0.34
	mNLDN	645	6.44	9.17	**7.2**	**75.1**	**2.7**	**42.4**	0.34
	hNLDN	645	6.44	9.18	7.2	75.3	2.7	42.6	**0.34**
	pNLDN	645	6.44	9.17	7.2	75.2	2.7	42.5	0.34
UM	Base	542	11.42	18.09	**18.7**	**82.7**	**6.7**	**58.4**	**0.58**
	mNLDN	542	11.42	18.10	18.7	82.8	6.7	58.5	**0.58**
	hNLDN	542	11.42	18.22	18.9	83.6	6.8	59.5	**0.58**
	pNLDN	542	11.42	18.09	18.7	**82.7**	**6.7**	58.4	**0.58**
LM	Base	1240	6.11	8.32	6.0	61.2	2.2	36.1	**0.68**
	mNLDN	1240	6.11	8.30	**6.0**	**61.1**	2.2	**35.9**	**0.68**
	hNLDN	1240	6.11	8.33	6.0	61.3	2.2	36.3	**0.68**
	pNLDN	1240	6.11	8.31	6.0	61.2	2.2	36.0	**0.68**
RM	Base	1370	3.90	4.00	3.7	60.0	**0.1**	**2.4**	0.58
	mNLDN	1370	3.90	4.01	**3.7**	**59.9**	**0.1**	2.6	0.58
	hNLDN	1370	3.90	4.02	3.7	60.0	0.1	3.3	**0.58**
	pNLDN	1370	3.90	4.00	3.7	60.0	**0.1**	**2.4**	0.58
PC	Base	2056	8.61	9.52	**9.1**	**62.8**	**0.9**	**10.6**	0.48
	mNLDN	2056	8.61	9.52	**9.1**	**62.8**	**0.9**	**10.6**	0.48
	hNLDN	2056	8.61	9.59	9.1	62.9	1.0	11.4	**0.48**
	pNLDN	2056	8.61	9.52	**9.1**	**62.8**	**0.9**	**10.6**	0.48

**Table 3.** Statistics of June–August 2011 accumulated precipitation (cm) and wet deposition of nitrate (${\text{NO}}_{3}^{-}$) for all model cases over the domain. The best performance metrics among the model cases are highlighted in bold.

Region	Case	Record	OBS (cm, kg ha^−1^)	MOD (cm, kg ha^−1^)	RMSE (cm, kg ha^−1^)	NME (%)	MB (cm, kg ha^−1^)	NMB (%)	*R*
Domain	precip	196	24.8	23.9	7.5	23	−0.9	−4	0.87
	Base	196	2.34	1.52	1.1	38	−0.8	−35	0.84
	mNLDN	196	2.34	1.98	**0.8**	**26**	−**0.4**	−**15**	**0.86**
	hNLDN	196	2.34	1.95	0.8	**26**	−**0.4**	−17	0.86
	pNLDN	196	2.34	1.68	1.0	33	−0.7	−28	0.85
NE	precip	31	38.6	35.9	9.5	19	−2.7	−7	0.79
	Base	31	2.96	2.32	1.1	29	−0.6	−23	0.70
	mNLDN	31	2.96	2.71	**0.9**	**24**	−0.3	−8	**0.76**
	hNLDN	31	2.96	2.74	**0.9**	**24**	−0.2	−**7**	0.74
	pNLDN	31	2.96	2.48	1.0	27	−0.5	−16	0.73
SE	precip	39	36.1	31.7	9.4	21	−4.3	−12	0.80
	Base	39	3.05	2.09	1.2	35	−1.0	−32	0.51
	mNLDN	39	3.05	2.97	**0.8**	**21**	−**0.1**	−**2**	**0.56**
	hNLDN	39	3.05	2.82	0.9	23	−0.2	−8	0.53
	pNLDN	39	3.05	2.43	1.0	27	−0.6	−20	0.54
UM	precip	45	28.8	26.1	6.8	20	−2.7	−9	0.51
	Base	45	3.17	1.98	1.4	38	−1.2	−38	0.73
	mNLDN	45	3.17	2.51	**0.9**	**24**	−**0.7**	−**21**	**0.77**
	hNLDN	45	3.17	2.48	**0.9**	25	−**0.7**	−22	**0.77**
	pNLDN	45	3.17	2.15	1.2	33	−1.0	−32	0.76
LM	precip	12	12.3	10.4	4.1	29	−2.0	−16	0.90
	Base	12	1.44	0.85	0.7	41	−0.6	−41	0.90
	mNLDN	12	1.44	1.16	**0.6**	33	−**0.3**	−**19**	0.88
	hNLDN	12	1.44	1.13	**0.6**	**32**	−**0.3**	−21	**0.89**
	pNLDN	12	1.44	0.93	0.7	36	−0.5	−35	0.88
RM	precip	50	13.7	18.2	6.9	39	4.4	32	0.91
	Base	50	1.63	0.8	1.0	51	−0.8	−51	0.90
	mNLDN	50	1.63	1.1	**0.7**	34	−**0.5**	−32	**0.91**
	hNLDN	50	1.63	1.12	**0.7**	**33**	−**0.5**	−**31**	0.90
	pNLDN	50	1.63	0.86	1.0	48	−0.8	−47	**0.91**
PC	precip	19	7.01	6.53	**2.4**	29	−**0.48**	−6.8	0.84
	Base	19	0.31	0.31	**0.18**	44	**0.00**	−1.0	0.88
	mNLDN	19	0.31	0.33	0.19	48	0.01	3.9	**0.89**
	hNLDN	19	0.31	0.33	0.20	50	0.02	6.6	**0.89**
	pNLDN	19	0.31	0.31	**0.18**	44	**0.00**	−**0.3**	0.88
